# Age- and Sex-Based Hematological and Biochemical Parameters for *Macaca fascicularis*

**DOI:** 10.1371/journal.pone.0064892

**Published:** 2013-06-10

**Authors:** Liang Xie, Fan Xu, Shigang Liu, Yongjia Ji, Qinming Zhou, Qingyuan Wu, Wei Gong, Ke Cheng, Juan Li, Leilei Li, Liang Fang, Linke Zhou, Peng Xie

**Affiliations:** 1 Institute of Neuroscience, Chongqing Medical University, Chongqing, China; 2 Chongqing Key Laboratory of Neurobiology, Chongqing, China; 3 Department of Neurology, The First Affiliated Hospital of Chongqing Medical University, Chongqing, China; 4 Suzhou Xishan Zhongke Laboratory Animal Co., Ltd., Suzhou, China; 5 Chinese Center for Disease Control and Prevention, Beijing, China; University of Alabama at Birmingham, United States of America

## Abstract

**Background:**

The cynomolgus monkey (*Macaca fascicularis*) has been increasingly used in biomedical research, making knowledge of its blood-based parameters essential to support the selection of healthy subjects and its use in preclinical research. As age and sex affect these blood-based parameters, it is important to establish baseline indices for these parameters on an age and sex basis and determine the effects of age and sex on these indices.

**Methods:**

A total of 917 cynomolgus monkeys (374 males and 543 females) were selected and segregated by age (five groups) and sex. A total of 30 hematological and 22 biochemical parameters were measured, and the effects of age and sex were analyzed.

**Results:**

Baseline indices for hematological and biochemical parameters were separately established by age and sex. Significant effects by age, sex, and age-sex interaction were observed in a number of blood parameters. In the 49–60 months and 61–72 months age groups, red blood cell count, hemoglobulin, and hematocrit showed significantly lower values (*P*<0.01) in females than males. Serum alkaline phosphatase varied with age in both sexes (*P*<0.01) and was significantly higher in females than males (*P*<0.05) in the groups aged 13–24 months and 25–36 months; however, in the three groups aged over 25–36 months, serum alkaline phosphatase was significantly lower in females than males (*P*<0.01). Creatinine concentration increased with age (*P*<0.01) in all age groups; specifically in the groups aged 49–60 months and 61–72 months, creatinine was significantly higher (*P*<0.01) in males than females. Total protein and globulin both increased with age (*P*<0.01).

**Conclusion:**

The baseline values of hematological and biochemical parameters reported herein establish reference indices of blood-based parameters in the cynomolgus monkey by age and sex, thereby aiding researchers in selecting healthy subjects and evaluating preclinical studies using this species.

## Introduction

The cynomolgus monkey (*Macaca fascicularis*), an Old World monkey, has been increasingly used in biomedical research. Old World monkeys, New World monkeys, apes, and humans (collectively termed simians) are grouped under the primate infraorder *Anthropoidea* on account of their larger and more complicated brains relative to those of other primates. The cynomolgus monkey appears to originate from tropical insular Southeast Asia. Its natural range extends southward to the Malay Peninsula, Sumatra, Borneo, Java and the Lesser Sunda Islands (including Bali and Timor), the Philippines, and westward to southern Burma, southern Bangladesh and India [1]. The species is most commonly observed at low elevations, where it prefers seashore, swamp, and mangrove forests and river banks. The typical life span of the cynomolgus monkey ranges from 25–30 years [2]. Sexual maturity is reached at four years of age for the female and six years of age for the male [3].

Cynomolgus monkeys have been increasingly used in research circles, as they are more anatomically and physiologically homologous to humans as compared to other animal models (e.g., rats, pigs) and share many other characteristics with humans. First, cynomolgus monkeys are sexually dimorphic with respect to size (males: 412–648 mm [excluding tail], 4.7–8.3 kg; females: 385–503 mm [excluding tail], 2.5 and 5.7 kg) [4]. Second, cynomolgus monkeys are omnivorous and consume a variety of foods (e.g., fruits, crabs, flowers, insects, leaves, fungi, grasses, and clay), reflecting the diverse habitats they occupy. Third, similar to human females, female cynomolgus monkeys exhibit menstrual cycles as the uterine lining periodically sloughs on a monthly basis (mean length: 28–32 days) and experience reproductive functional loss with age (e.g., progression from normal menstruation to irregular cycles to cycle cessation and infertility) [5]. Fourth, cynomolgus monkeys are susceptible to age-related pathologies commonly observed in humans, such as decreased cardiovascular function, functional cognitive decline and biochemical changes similar to those found in Alzheimer’s disease [6], [7], bone loss [8], knee osteoarthritis [9], obesity, and diabetes [10] (and complications therefrom, including diabetic neuropathy and retinopathy). Fifth, cynomolgus monkeys are natural hosts to a wide variety of viral agents (e.g., Herpes B [11], simian retroviruses [12], ebola-related virus, hepatitis A virus [13], simian parvovirus [14], measles [15], and monkeypox [16] ) with analogs in humans.

As a result of the increased use of the cynomolgus monkey, it has become imperative to assess the hematological and biochemical parameters of this monkey species in order to support the selection of healthy subjects, as well as to build reference indices for their use in preclinical research (e.g., transplantation, toxicology). In order to build an integrated, high-quality foundation for future cynomolgus monkey-based research, one of the first and most important investigations should be to assess and establish comprehensive and accurate reference indices of hematological and biochemical parameters for the species.

Although there have been some published studies reporting hematological and biochemical values of cynomolgus monkeys, the sample size of most of these studies has been relatively small [17], [18]. Furthermore, some prior studies have reported the values combined for all age groups [19]. In addition, some valuable indices have never been reported before [20], [21] (e.g., immature reticulocyte fraction, low fluorescence reticulocyte percentage). To our knowledge, the values of hematological and serum biochemical parameters in non-human primates may be influenced by age [22], [23], sex [24], [25], body weight [26], species [27], [28], anaesthetics [29]–[31], breeding background [18], gravidity [32], [33], and disease [34], [35]; among these factors, age and sex are important for comparatively evaluating experimental data. Therefore, the aim of this study is to establish comprehensive and accurate reference indices of 52 hematological and biochemical parameters by age and sex based on 917 cynomolgus monkeys and determine the effects of age and sex on these blood values, thereby providing researchers with improved assessment criteria in selecting healthy subjects and evaluating preclinical studies using this macaque species.

## Materials and Methods

### Subjects and Ethical Statement

The study population was composed of 917 cynomolgus monkeys (374 males and 543 females; age: 13–72 months) obtained from Xishan Zhongke Laboratory Animals Co. Ltd. (Suzhou, China) (Table 1). Animals were kept at the Suzhou Xishan Zhongke Laboratory Animal Co., Ltd., which is accredited by the Association for the Assessment and Accreditation of Laboratory Animal Care International (AAALAC).

**Table 1:** Sample sizes by age and sex.

Age (months)	Males and females (n)	Males *(*n)	Females (n)
13–24	324	162	162
25–36	299	121	178
37–48	88	16	72
49–60	115	31	84
61–72	91	44	47
Total	917	374	543

All subjects were housed in same-sex social groups that were formed when the monkeys were six months of age and were stable thereafter. The groups were housed in indoor pens measuring 8 m × 3 m × 3 m (L×W×H), given water *ad libitum*, and fed daily with fresh fruits, vegetables, and compound high-nutrition monkey food. Indoor pens were maintained at a temperature higher than 18°C and relative humidity of 40%–70%. The living environment and animal care procedures were detailed in a previous report [36], which were in accordance with Chinese regulatory requirements and the Association for the Assessment and Accreditation of Laboratory Animal Care International (AAALAC) guidelines. These monkeys were determined to be healthy by history and veterinary examination, and they were free of tuberculosis and Herpes B virus. All procedures involving macaques were approved by the Animal Care and Use Committee of Chongqing Medical University (Approval No: 20100031) and were in compliance with the Guide for the Care and Use of Laboratory Animals [37]. Blood samples were collected during biannual routine health check-ups. No animals were sacrificed in the study.

### Blood Sample Collection and Preparation

After overnight fasting (14–16 h), conscious monkeys were restrained humanely by experienced animal care technicians, and 4-ml blood samples were drawn from cephalic veins through 22-gauge needles. Then, 2-ml aliquots from all blood samples were individually transferred into ethylene diamine tetraacetic acid-potassium (EDTA-K2) tubes for hematological analysis; the remaining 2-ml aliquots were stored in plastic tubes without anti-coagulants for biochemical analysis. The latter aliquots were allowed to clot at room temperature for 30–60 min, and the serum was separated by centrifugation at 1600 g for 15 min. The serum was stored in polypropylene tubes at −20°C until later analysis.

### Hematological and Biochemical Analysis

Hematological analysis was performed using a Sysmex XT-2000iv automated hematology analyzer (Sysmex Corporation, Kobe, Japan). Biochemical analysis was carried out using an Architect c8000 analyzer (Abbott Laboratories, Abbott Park, USA). Hematological and biochemical parameters are listed in Table 2.

**Table 2:** List of hematological and biochemical parameters*.

Parameter	Abbreviation	Unit
Red blood cell	RBC	10^12^/l
Hemoglobin	HGB	g/l
Hematocrit	HCT	%
Mean corpuscular volume	MCV	fl
Mean corpuscular hemoglobin	MCH	pg
Mean corpuscular hemoglobin concentration	MCHC	g/l
Red blood cell volume distribution width-SD	RDW-SD	–
Red blood cell volume distribution width-CV	RDW-CV	%
Reticulocyte	RET	10^9^/l
Reticulocyte percentage	RET%	%
High fluorescence reticulocyte percentage	HFR	%
Median fluorescence reticulocyte percentage	MFR	%
Low fluorescence reticulocyte percentage	LFR	%
Immature reticulocyte fraction	IRF	%
White blood cell	WBC	10^9^/l
Neutrophil	NEUT	10^9^/l
Neutrophil percentage	NEUT%	%
Basophil	BASO	10^9^/l
Basophil percentage	BASO%	%
Eosinophil	EO	10^9^/l
Eosinophil percentage	EO%	%
Lymphocyte	LYMPH	10^9^/l
Lymphocyte percentage	LYMPH%	%
Monocyte	MONO	10^9^/l
Monocyte percentage	MONO%	%
Platelet	PLT	10^9^/l
Mean platelet volume	MPV	fl
Plate volume distribution width	PDW	%
Platelet large cell ratio	P-LCR	%
Plateletcrit	PCT	%
Total bilirubin	TBIL	µmol/l
Total protein	TP	g/l
Albumin	ALB	g/l
Globulin	GLOB	g/l
Albumin/globulin ratio	A/G	–
Alanine aminotransferase	ALT	IU/L
Aspartate aminotransferase	AST	IU/L
Alkaline phosphatase	ALP	IU/L
Gamma glutamyltransferase	GGT	IU/L
Lactate dehydrogenase	LDH	IU/L
Creatine kinase	CK	IU/L
Blood urea nitrogen	BUN	mmol/l
Creatinine	CREA	µmol/l
Glucose	GLU	mmol/l
Triglyceride	TG	mmol/l
Total cholesterol	TCHOL	mmol/l
Potassium	K	mmol/l
Sodium	Na	mmol/l
Chloride	Cl	mmol/l
Calcium	Ca	mmol/l
Phosphorus	P	mmol/l
Magnesium	Mg	mmol/l

* The first 30 items are hematological parameters; the latter 22 items are biochemical parameters.

### Statistical Analysis

All data, including hematological and biochemical references, were presented as means ± standard errors (Tables S1–S10). The two-way unbalanced analysis of variance (ANOVA) was used to examine the effect of sex, age, and sex-age interaction (Tables 3, 4). Moreover, the post hoc test (by one-way ANOVA) was executed to distinguish the differences in the five age groups within the same sex. Finally, a Student’s *t*-test was applied to detect significant differences between male and female subjects in the same age groups. The significance level (α) was set to 0.05. All data processing, data management, and statistical analysis were performed using Stata (College station, Texas, USA.12.0).

**Table 3:** Summary of age and sex effects on hematological parameters.

Parameter	Age	Sex	Interaction
RBC	F(4,907) = 5.23, *P*<0.01	F(1,907) = 35.26, *P*<0.01	F(4,907) = 9.15, *P*<0.01
HGB	F(4,907) = 3.16, *P*<0.05	F(1,907) = 32.18, *P*<0.01	F(4,907) = 9.28, *P*<0.01
HCT	F(4,907) = 4.42, *P*<0.05	F(1,907) = 20.34, *P*<0.01	F(4,907) = 11.34, *P*<0.01
MCV	F(4,907) = 9.50, *P*<0.01	F(1,907) = 10.24, *P*<0.05	F(4,907) = 2.08, NS
MCH	F(4,907) = 2.31, NS	F(1,907) = 1.04, NS	F(4,907) = 1.40, NS
MCHC	F(4,907) = 9.35, *P*<0.01	F(1,907) = 14.59, *P*<0.01	F(4,907) = 0.23, NS
RDW-SD	F(4,907) = 3.26, *P*<0.05	F(1,907) = 0.01, NS	F(4,907) = 1.62, NS
RDW-CV	F(4,907) = 2.10, NS	F(1,907) = 3.07, NS	F(4,907) = 0.66, NS
RET	F(4,907) = 1.45, NS	F(1,907) = 18.76, *P*<0.01	F(4,907) = 3.14, *P*<0.05
RET%	F(4,907) = 2.72, *P*<0.05	F(1,907) = 25.69, *P*<0.01	F(4,907) = 3.97, *P*<0.01
HFR	F(4,907) = 2.77, *P*<0.05	F(1,907) = 9.10, *P*<0.05	F(4,907) = 2.43, *P*<0.05
MFR	F(4,907) = 3.75, *P*<0.05	F(1,907) = 13.78, *P*<0.01	F(4,907) = 1.10, NS
LFR	F(4,907) = 1.04, NS	F(1,907) = 1.46, NS	F(4,907) = 1.57, NS
IRF	F(4,907) = 1.04, NS	F(1,907) = 1.46, NS	F(4,907) = 1.57, NS
WBC	F(4,907) = 1.05, NS	F(1,907) = 6.10, *P*<0.05	F(4,907) = 2.56, *P*<0.05
NEUT	F(4,907) = 13.66, *P*<0.01	F(1,907) = 39.59, *P*<0.01	F(4,907) = 1.36, NS
NEUT%	F(4,907) = 22.29, *P*<0.01	F(1,907) = 47.89, *P*<0.01	F(4,907) = 3.66, *P*<0.01
BASO	F(4,907) = 11.92, *P*<0.01	F(1,907) = 3.73, NS	F(4,907) = 2.93, *P*<0.05
BASO%	F(4,907) = 14.34, *P*<0.01	F(1,907) = 0.53, NS	F(4,907) = 1.28, NS
EO	F(4,907) = 9.35, *P*<0.01	F(1,907) = 1.05, NS	F(4,907) = 1.28, NS
EO%	F(4,907) = 8.26, *P*<0.01	F(1,907) = 0.01, NS	F(4,907) = 0.30, NS
LYMPH	F(4,907) = 15.16, *P*<0.01	F(1,907) = 15.94, *P*<0.01	F(4,907) = 5.49, *P*<0.01
LYMPH%	F(4,907) = 23.66, *P*<0.01	F(1,907) = 45.85, *P*<0.01	F(4,907) = 3.66, *P*<0.01
MONO	F(4,907) = 2.43, *P*<0.05	F(1,907) = 1.81, NS	F(4,907) = 4.01, *P*<0.01
MONO%	F(4,907) = 2.08, NS	F(1,907) = 11.07, *P*<0.01	F(4,907) = 1.46, NS
PLT	F(4,907) = 0.16, NS	F(1,907) = 0.02, NS	F(4,907) = 2.05, NS
MPV	F(4,907) = 3.18, *P*<0.05	F(1,907) = 3.58, NS	F(4,907) = 2.15, NS
PDW	F(4,907) = 2.64, *P*<0.05	F(1,907) = 1.01, NS	F(4,907) = 1.03, NS
P-LCR	F(4,907) = 4.11, *P*<0.01	F(1,907) = 6.44, *P*<0.05	F(4,907) = 2.58, *P*<0.05
PCT	F(4,907) = 0.41, NS	F(1,907) = 1.12, NS	F(4,907) = 2.28, NS

NS, not significant.

**Table 4:** Summary of age and sex effects on biochemical parameters.

Parameter	Age	Sex	Interaction
BILT	F(4,907) = 2.15, NS	F(1,907) = 1.89, NS	F(4,907) = 0.25, NS
TP	F(4,907) = 23.89, *P*<0.01	F(1,907) = 0.02, NS	F(4,907) = 2.31, NS
ALB	F(4,907) = 3.01, *P*<0.05	F(1,907) = 6.68, *P*<0.01	F(4,907) = 4.64, *P*<0.01
GLOB	F(4,907) = 71.87, *P*<0.01	F(1,907) = 7.89, *P*<0.01	F(4,907) = 3.04, *P*<0.05
A/G	F(4,907) = 44.02, *P*<0.01	F(1,907) = 11.24, *P*<0.01	F(4,907) = 5.24, *P*<0.01
ALT	F(4,907) = 16.62, *P*<0.01	F(1,907) = 0.00, NS	F(4,907) = 3.49, *P*<0.01
AST	F(4,907) = 45.88, *P*<0.01	F(1,907) = 3.09, NS	F(4,907) = 2.36, NS
ALP	F(4,907) = 112.80, *P*<0.01	F(1,907) = 57.01, *P*<0.01	F(4,907) = 26.50, *P*<0.01
GGT	F(4,907) = 17.90, *P*<0.01	F(1,907) = 9.62, *P*<0.01	F(4,907) = 2.83, *P*<0.05
LDH	F(4,907) = 75.66, P<0.01	F(1,907) = 10.29, *P*<0.01	F(4,907) = 2.68, *P*<0.05
CK	F(4,907) = 3.34, *P*<0.01	F(1,907) = 0.35, NS	F(4,907) = 0.07, NS
BUN	F(4,907) = 49.73, *P*<0.01	F(1,907) = 0.14, NS	F(4,907) = 1.32, NS
CREA	F(4,907) = 289.64, *P*<0.01	F(1,907) = 43.66, *P*<0.01	F(4,907) = 21.33, *P*<0.01
GLU	F(4,907) = 14.46, *P*<0.01	F(1,907) = 0.39, NS	F(4,907) = 2.26, NS
TG	F(4,907) = 4.25, *P*<0.01	F(1,907) = 0.03, NS	F(4,907) = 1.43, NS
TCHOL	F(4,907) = 2.89, *P*<0.05	F(1,907) = 3.33, NS	F(4,907) = 2.55, *P*<0.05
K	F(4,907) = 12.61, *P*<0.01	F(1,907) = 0.09, NS	F(4,907) = 5.54, *P*<0.01
Na	F(4,907) = 29.18, *P*<0.01	F(1,907) = 0.04, NS	F(4,907) = 6.91, *P*<0.01
Cl	F(4,907) = 0.86, NS	F(1,907) = 23.76, *P*<0.01	F(4,907) = 2.22, NS
Ca	F(4,907) = 9.12, *P*<0.01	F(1,907) = 5.02, *P*<0.05	F(4,907) = 8.68, *P*<0.01
P	F(4,907) = 5.21, *P*<0.01	F(1,907) = 23.46, *P*<0.01	F(4,907) = 2.51, *P*<0.05
Mg	F(4,907) = 14.85, *P*<0.01	F(1,907) = 0.82, NS	F(4,907) = 4.10, *P*<0.01

NS, not significant.

## Results

### Reference Ranges of Hematological and Biochemical Values

Reference values and ranges of hematological and biochemical values by sex and age are reported in Tables S1–S10.

### Effects of Age and Sex on Hematological Values

**Figure 1:**
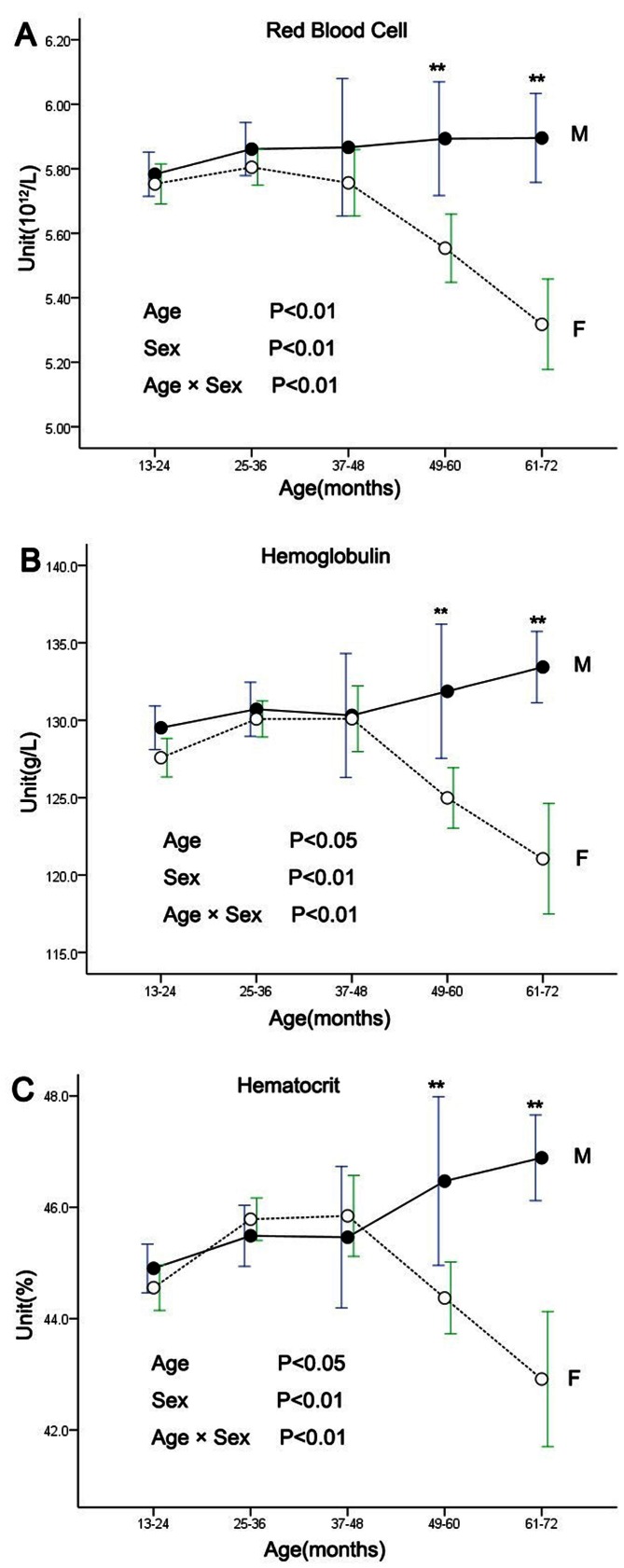
Changes of red blood cells (A), hemoglobulin (B), hematocrit (C) in males and females with age. Data are presented as means ± SDs at each point. Significant difference between indicated age groups as reveled by *post hoc* analyses (*P<0.05, **P<0.01).

The results of analyzing age, sex and sex-age interaction on hematological values are presented in Table 3. Significant effects by age were shown for RBC, HGB, HCT, MCV, MCHC, RDW-SD, RET%, HFR, MFR, NEUT, NEUT%, BASO, BASO%, EO, EO%, LYMPH, LYMPH%, MONO, MPV, PDW, and P-LCR. Significant effects by sex were observed for RBC, HGB, HCT, MCV, MCHC, RET, RET%, HFR, MFR, WBC, NEUT, NEUT%, LYMPH, LYMPH%, MONO%, and P-LCR. Significant effects by age-sex interaction were found for RBC, HGB, HCT, RET, RET%, HFR, WBC, NEUT%, BASO, LYMPH, LYMPH%, MONO, and P-LCR. For the 49–60 months and 61–72 months age groups, RBC, HGB, and HCT showed significantly lower values (*P*<0.01) in females than males (Fig. 1).

### Effect of Age and Sex on Biochemical Values

**Figure 2:**
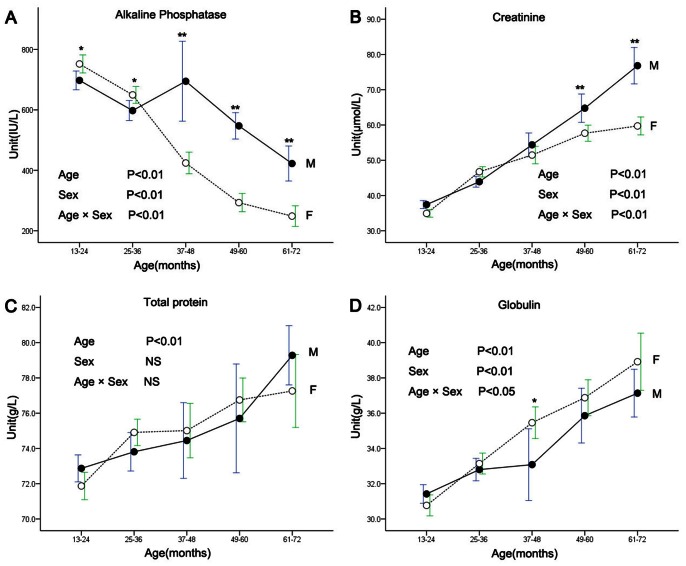
Changes of alkaline phosphatase (A), creatinine (B), total protein (C) and globulin (D) in males and females with age. Data are presented as means ± SDs at each point. Significant difference between indicated age groups as reveled by *post hoc* analyses (*P<0.05, **P<0.01). NS, not significant.

The results of analyzing age, sex, and sex-age interaction on biochemical values are listed in Table 4, Significant effects by age were shown for TP, ALB, GLOB, A/G, ALT, AST, ALP, GGT, LDH, CK, BUN, CREA, GLU, TG, TCHOL, K, Na, Ca, P, and Mg. Significant effects by sex were shown for ALB, GLOB, A/G, ALP, GGT, LDH, CREA, Cl, Ca, and P. Significant effects by age-sex interaction were shown for ALB, GLOB, A/G, ALT, ALP, GGT, LDH, CREA, TCHOL, K, Na, Ca, P, and Mg. Serum ALP varied with age in both sexes (*P*<0.01) and was significantly higher in females than males (*P*<0.05) in the groups aged 13–24 months and 25–36 months; however, in the three groups aged over 25–36 months, serum ALP was significantly lower in females than males (*P*<0.01) (Fig. 2A). CREA concentration increased with age (*P*<0.01) in all age groups; specifically in the groups aged 49–60 months and 61–72 months, CREA was significantly higher (*P*<0.01) in males than females (Fig. 2B). Total protein and globulin increase with age (*P*<0.01, Figs. 2C, 2D).

## Discussion

In this study, hematological and biochemical analyses of 917 cynomolgus monkeys were performed in order to establish baseline reference indices for the macaque species by age and sex and determine the effects of age and sex on these blood indices. Reference indices are essential to evaluating preclinical findings and selecting healthy subjects. Due to its strong homology with the human, the cynomolgus monkey serves as a valuable animal model; for example, the species already plays an important role in aging disease, virological, reproductive physiological, behavioral, and neurophysiological research.

As previous reports have shown age and sex to be factors affecting hematological and biochemical parameters in non-human primates, it is essential to construct reference indices of blood-based parameters across different age groups by sex. In comparison to prior studies that have constructed reference ranges in cynomolgus monkeys [17], [21], most blood indices showed remarkable similarity, while some parameters (e.g., RBC, GLOB, AST and LDH) displayed large inter-study variability in the same age range. This discrepancy may be due, in part, to the differing geographic origins of the monkeys under investigation. In addition, these results provide valuable supplementation to some scantly-studied parameters (e.g., IRF, HFR, MFR, and LFR).

In addition to age and sex, hematological and biochemical parameters of non-human primates can also be affected by other factors, such as species, anaesthetics, fasting, gravidity, etc. Thus, several experimental conditions should be taken into account when comparatively interpreting results from other studies. First, blood sample collection was performed on conscious animals in our study, as conscious collection may better reflect actual physiological values; accordingly, prior reports have demonstrated that anaesthetics can alter serum biochemical and hematological variables. For example, ketamine anesthesia for cynomolgus monkeys produces reductions in the white blood cell count, glucose, and potassium, along with increases in aspartate aminotransferase and creatine phosphokinase as compared to the conscious state [29]; moreover, similar findings have been reported in rhesus [30] and bonnet monkeys [31]. Second, all subjects in our study were fasting 14–16 hours before blood collection; according to the recommendations of the American Association for Clinical Chemistry’s Division of Animal Clinical Chemistry (AACC-DACC) and the American Society for Veterinary Clinical Pathology’s (ASVCP) Joint Committee on Clinical Pathology Testing of Laboratory Species, a 12–18 hour overnight fast for animal species prior to blood collection is essential [38], [39]. As drug absorption rates are known to be influenced by fed and fasting states [40], [41], the rationale of using fasting animals is to reduce variability in analytes (e.g., glucose, triglycerides), which are highly sensitive to fed and fasting states [42]. Moreover, all subjects in this study were housed in social groups to mimic wild conditions, so they were allowed to take food freely. Therefore, food intake was not controlled. As some analytes can vary due to differential consumption, we choose to collect blood after overnight fasting in order to reduce the variability and standardize analyte concentrations. One prior study [43] has reported on the effects of fasting in cynomolgus monkeys and demonstrated that several indices (e.g. BUN, ALP, AST) do vary between fed and fasting states; however, the health of the animals was not affected. Thus, when comparing results, food intake should be considered. Third, it is necessary to consider inter-species variability when assessing blood-based parameters from other non-human primate species. Chen et al. [27] has established reference values for hematological and biochemical parameters of Chinese rhesus monkeys aged 3–5 years. When contrasted to the parameters of the groups aged 37–48 months and 49–60 months here, GLOB levels of cynomolgus monkeys were 1.45- and 1.51-fold higher than rhesus monkeys, respectively. Moreover, TBIL levels of rhesus monkeys were 1.42- and 1.52-fold higher than cynomolgus monkeys, respectively. However, several parameters displayed similarities between the two species (e.g., ALP, LDH, K, Na, Ca, Mg, and Cl). Therefore, although both monkeys are macaque species, the cynomolgus monkey and rhesus monkey display a high degree of inter-species variability in their blood-based parameters. Fourth, the animals in this study were non-pregnant, but differences in hematological and biochemical parameters between pregnant and non-pregnant monkeys have been previously reported [32], [33], [44], [45]. If the reference indices presented here are applied to other studies,the experimental conditions should be consistent with those described above; otherwise, the presumed “normal” values may be beyond the normal range.

Age- and sex-related differences were apparent in several hematological and biochemical parameters here. Red blood cell count, hemoglobulin, and hematocrit are key clinical diagnostic indices for anemia and internal haemorrhage. Consistent with our findings in the two groups aged over 37–48 months, previous data from various non-human primate species [21], [46], [47] and humans [48] have demonstrated that females display significantly lower values of RBC, HGB, and HCT than males. This phenomenon may relate to menstruation-related blood loss.

Serum alkaline phosphatase (ALP) is a commonly-used biochemical marker reflecting bone formation and osteoblast activity [49]. This study found that serum ALP declines with age, which may be attributable to skeletal growth velocity. Decreases in ALP concentrations have also been observed in the bonnet monkey, marmoset, and African green monkey [28], [50], [51]. Interestingly, sex-based differences were also found here. In the 13–24 month and 25–36 month age groups, serum ALP concentrations were higher in females than males; however, the situation was reversed in the 37–72 month age group. This may be related to different development time courses between males and females; specifically, the growth development period of male cynomolgus monkeys is longer than females and usually ends at 5 to 6 years of age. Similar changes in ALP have also been reported in vervet monkeys [46] and humans [52].

Creatinine (CREA) is a low-molecular weight nitrogen compound produced by muscle metabolism, and elevated CREA levels are known to occur with greater muscle mass ratios under normal conditions. In this study, the CREA level was found to be elevated with age and to be significantly higher in males than females in the 49–60 month and 61–72 month age groups, probably on account of the greater muscle mass in these subjects. This observation is consistent with other non-human primate species [25], [46], [50], [53]; however, Annarita et al. [54] reported no significant difference in CREA levels between juveniles and adults. In addition, total protein and globulin showed age-related differences here, which were also found in the rhesus monkey [27], bonnet monkey [50] and chimpanzee [55]. This phenomenon may be related to developmental characteristics.

Considering that all subjects in this study were under 72 months, blood-based reference indices for cynomolgus monkeys older than 72 months of age should be supplemented in future studies with the goal of constructing a more holistic database of reference indices while determining the effects of age and sex across a wider span of age groups.

### Conclusion

In conclusion, the baseline values of hematological and biochemical parameters reported herein establish comprehensive and accurate reference indices of hematological and biochemical parameters in the cynomolgus monkey by age and sex, thereby providing researchers with improved assessment criteria in selecting healthy subjects and evaluating preclinical studies using this macaque species.

## Supporting Information

Table S1 **Hematological values and ranges of cynomolgus monkeys aged 13–24 months.**
(DOC)

Table S2 **Hematological values and ranges of cynomolgus monkeys aged 25–36 months.**
(DOC)

Table S3 **Hematological values and ranges of cynomolgus monkeys aged 37–48 months.**
(DOC)

Table S4 **Hematological values and ranges of cynomolgus monkeys aged 49–60 months.**
(DOC)

Table S5 **Hematological values and ranges of cynomolgus monkeys aged 61–72 months.**
(DOC)

Table S6 **Biochemical values and ranges of cynomolgus monkeys aged 13–24 months.**
(DOC)

Table S7 **Biochemical values and ranges of cynomolgus monkeys aged 25–36 months.**
(DOC)

Table S8 **Biochemical values and ranges of cynomolgus monkeys aged 37–48 months.**
(DOC)

Table S9 **Biochemical values and ranges of cynomolgus monkeys aged 49–60 months.**
(DOC)

Table S10 **Biochemical values and ranges of cynomolgus monkeys aged 61–72 months.**
(DOC)
