# Supplementary material for: Age- and Sex-Based Hematological and Biochemical Parameters for Macaca fascicularis
Source: PLoS One. 2013 Jun 10;8(6):e64892. doi: 10.1371/journal.pone.0064892 (PMC3677909; doi:10.1371/journal.pone.0064892)
Supplement: Table S1 — Hematological values and ranges of cynomolgus monkeys aged 13–24 months. (DOC) [file pone.0064892.s001.doc]

**Table S1. Hematological values and ranges of cynomolgus monkeys aged 13-24 months.***

| **Parameter (unit)** | **Males and**  **females (n=324)** | **Males**  **(n=162)** | **Females (n=162)** | **Male range (n=162)** | **Female range (n=162)** |
| --- | --- | --- | --- | --- | --- |
| Red blood cell (10^12^/l) | 5.77±0.42 | 5.78±0.44 | 5.75±0.40 | 4.90-6.66 | 4.95-6.75 |
| Hemoglobulin (g/l) | 128.55±8.50 | 129.51±8.95 | 127.58±7.94 | 111.61-147.41 | 111.70-143.46 |
| Hematocrit (%) | 44.73±2.70 | 44.90±2.79 | 44.56±2.61. | 39.32-49.69 | 39.34-49.78 |
| Mean corpuscular volume (fl) | 77.71±4.07 | 77.82±4.13 | 77.60±4.01 | 69.56-86.08 | 69.58-85.62 |
| Mean corpuscular hemoglobulin (pg) | 22. 32±1.15 | 22.43±1.11 | 22.21±1.19 | 20.21-24.65 | 19.83-24.59 |
| Mean corpuscular hemoglobulin concentration (g/l) | 287.49±7.14 | 288.47±7.56 | 286.50±6.59 | 273.35-303.59 | 273.32-299.68 |
| Red blood cell volume distribution width-SD | 37.08±2.65 | 37.34±3.01 | 36.82±2.22 | 31.32-43.46 | 32.38-41.26 |
| Red blood cell volume distribution width-CV (%) | 13.31±1.00 | 13.38±0.97 | 13.25±1.02 | 11.44-15.32 | 11.21-15.29 |
| Reticulocyte (10^9^/l) | 53.61±24.23 | 54.12±25.39 | 53.09±23.08 | 9.60-104.90 | 10.30-99.25 |
| Reticulocyte percentage (%) | 0.93±0.44 | 0.94±0.47 | 0.93±0.42 | 0.18-1.88 | 0.20-1.77 |
| High fluorescence reticulocyte percentage (%) | 10.59±6.80 | 10.52±6.31 | 10.66±7.28 | 1.30-23.14 | 1.30-25.22 |
| Median fluorescence reticulocyte percentage (%) | 5.34±3.02 | 5.10±2.75 | 5.57±3.26 | 0-10.60 | 0-12.09 |
| Low fluorescence reticulocyte percentage (%) | 84.07±7.43 | 84.38±6.90 | 83.77±7.94 | 70.58-98.18 | 67.89-99.65 |
| Immature reticulocyte fraction (%) | 15.93±7.43 | 15.63±6.90 | 16.23±7.94 | 1.83-29.43 | 0.35-32.11 |
| White blood cell (10^9^/l) | 13.03±3.02 | 12.73±2.16 | 13.33±3.66 | 8.41-17.05 | 6.01-20.65 |
| Neutrophil (10^9^/l) | 4.67±2.65 | 4.20±1.61 | 5.16±3.32 | 0.98-7.42 | 1.69-11.80 |
| Neutrophil percentage (%) | 35.32±13.34 | 33.05±10.95 | 37.59±15.07 | 11.15-54.95 | 7.45-67.73 |
| Basophil (10^9^/l) | 0.03±0.02 | 0.03±0.01 | 0.03±0.20 | 0.01-0.05 | 0-0.07 |
| Basophil percentage (%) | 0.16±0.08 | 0.16±0.07 | 0.16±0.08 | 0.02-0.30 | 0-0.32 |
| Eosinophil (10^9^/l) | 0.23±0.22 | 0.22±0.23 | 0.23±0.20 | 0.01-0.68 | 0-0.63 |
| Eosinophil percentage (%) | 1.33±1.20 | 1.34±1.36 | 1.31±1.03 | 0.10-4.06 | 0-3.37 |
| Lymphocyte (10^9^/l) | 7.28±2.22 | 7.43±1.96 | 7.14±2.44 | 3.51-11.35 | 2.26-12.02 |
| Lymphocyte percentage (%) | 56.52±12.93 | 58.35±11.19 | 54.70±14.26 | 35.97-80.73 | 26.18-83.22 |
| Monocyte (10^9^/l) | 0.85±0.29 | 0.89±0.29 | 0.82±0.29 | 0.31-1.47 | 0.24-1.40 |
| Monocyte percentage (%) | 6.67±1.95 | 7.10±2.09 | 6.25±1.72 | 2.92-11.28 | 2.81-9.69 |
| Platelet (10^9^/l) | 375.32±89.79 | 381.01±94.65 | 369.64±84.57 | 191.71-570.31 | 200.50-538.78 |
| Mean platelet volume (fl) | 12.66±1.10 | 12.63±1.13 | 12.68±1.07 | 10.37-14.89 | 10.54-14.82 |
| Plate volume distribution width (%) | 15.71±2.56 | 15.63±2.68 | 15.79±2.43 | 10.27-20.99 | 10.93-20.65 |
| Platelet large cell ratio (%) | 45.46±7.88 | 44.94±8.02 | 45.99±7.73 | 28.90-60.98 | 30.53-61.45 |
| Plateletcrit (%) | 0.47±0.09 | 0.48±0.10 | 0.46±0.09 | 0.28-0.68 | 0.28-0.64 |

^*^To exclude outliers, the range limits have been defined as 2×SD above and below the mean. Where the lower limit falls below zero, the lowest observed value was used.
