# Supplementary material for: Age- and Sex-Based Hematological and Biochemical Parameters for Macaca fascicularis
Source: PLoS One. 2013 Jun 10;8(6):e64892. doi: 10.1371/journal.pone.0064892 (PMC3677909; doi:10.1371/journal.pone.0064892)
Supplement: Table S2 — Hematological values and ranges of cynomolgus monkeys aged 25–36 months. (DOC) [file pone.0064892.s002.doc]

**Table S2. Hematological values and ranges of cynomolgus monkeys aged 25-36 months.***

| **Parameter (unit)** | **Males and females (n=299)** | **Males (n=121)** | **Females (n=178)** | **Male range (n=121)** | **Female range (n=178)** |
| --- | --- | --- | --- | --- | --- |
| Red blood cell (10^12^/l) | 5.83±0.41 | 5.86±0.45 | 5.80±0.37 | 4.96-6.76 | 5.06-6.54 |
| Hemoglobulin (g/l) | 130.34±8.53 | 130.71±9.59 | 130.08±7.75 | 111.53-149.89 | 114.58-145.58 |
| Hematocrit (%) | 45.67±2.75 | 45.49±3.01 | 45.79±2.55 | 39.47-51.51 | 40.69-50.89 |
| Mean corpuscular volume (fl) | 78.50±3.69 | 77.76±3.62 | 79.00±3.67 | 70.52-85.00 | 71.66-86.34 |
| Mean corpuscular hemoglobulin (pg) | 22.40±1.13 | 22.33±1.08 | 22.44±1.17 | 20.17-24.49 | 20.10-24.78 |
| Mean corpuscular hemoglobulin concentration (g/l) | 285.59±8.29 | 287.45±7.50 | 284.33±8.58 | 272.45-302.45 | 267.17-301.49 |
| Red blood cell volume distribution width-SD | 36.89±2.22 | 36.66±2.22 | 37.05±2.21 | 32.22-41.10 | 32.63-41.47 |
| Red blood cell volume distribution width-CV (%) | 13.08±0.87 | 13.12±0.88 | 13.05±0.87 | 11.36-14.88 | 11.31-14.79 |
| Reticulocyte (10^9^/l) | 51.82±24.83 | 47.06±17.33 | 55.06±28.43 | 12.40-81.72 | 18.7-111.92 |
| Reticulocyte percentage (%) | 0.89±0.46 | 0.80±0.29 | 0.96±0.54 | 0.22-1.38 | 0.30-2.04 |
| High fluorescence reticulocyte percentage (%) | 12.01±6.44 | 12.41±6.68 | 11.73±6.27 | 2.40-25.77 | 2.50-24.27 |
| Median fluorescence reticulocyte percentage (%) | 4.71±2.66 | 4.45±2.21 | 4.88±2.92 | 0.03-8.87 | 0-10.72 |
| Low fluorescence reticulocyte percentage (%) | 83.29±6.95 | 83.14±7.12 | 83.38±6.86 | 68.90-97.38 | 69.66-97.10 |
| Immature reticulocyte fraction (%) | 16.71±6.95 | 16.86±7.12 | 16.61±6.86 | 2.62-31.10 | 2.89-30.33 |
| White blood cell (10^9^/l) | 13.23±3.17 | 12.50±2.69 | 13.73±3.37 | 7.12-17.88 | 6.99-20.47 |
| Neutrophil (10^9^/l) | 4.38±2.13 | 3.85±1.71 | 4.74±2.32 | 0.43-7.27 | 0.10-9.38 |
| Neutrophil percentage (%) | 32.97±12.83 | 31.07±12.27 | 34.26±13.06 | 6.53-55.61 | 8.14-60.38 |
| Basophil (10^9^/l) | 0.03±0.02 | 0.02±0.01 | 0.03±0.02 | 0.01-0.04 | 0.01-0.07 |
| Basophil percentage (%) | 0.14±0.07 | 0.13±0.06 | 0.15±0.07 | 0.01-0.25 | 0.01-0.29 |
| Eosinophil (10^9^/l) | 0.36±0.34 | 0.32±0.28 | 0.39±037 | 0-0.88 | 0.02-1.13 |
| Eosinophil percentage (%) | 2.00±1.84 | 1.90±1.67 | 2.07±1.95 | 0-5.24 | 0.10-5.97 |
| Lymphocyte (10^9^/l) | 7.65±2.41 | 7.53±2.38 | 7.74±2.44 | 2.77-12.29 | 2.86-12.62 |
| Lymphocyte percentage (%) | 57.99±12.25 | 59.95±11.98 | 56.65±12.28 | 35.99-83.91 | 32.09-81.21 |
| Monocyte (10^9^/l) | 0.91±0.37 | 0.85±0.31 | 0.94±0.39 | 0.23-1.47 | 0.16-1.72 |
| Monocyte percentage (%) | 6.90±2.21 | 6.94±2.45 | 6.87±2.04 | 2.04-11.84 | 2.79-10.95 |
| Platelet (10^9^/l) | 369.75±98.06 | 386.46±116.29 | 358.38±81.86 | 153.88-619.04 | 194.66-522.10 |
| Mean platelet volume (fl) | 12.97±1.08 | 12.75±1.13 | 13.12±1.02 | 10.49-15.01 | 11.08-15.16 |
| Plate volume distribution width (%) | 16.28±2.50 | 15.85±2.61 | 16.57±2.39 | 10.63-21.07 | 11.79-21.35 |
| Platelet large cell ratio (%) | 48.00±7.60 | 45.90±7.98 | 49.43±7.00 | 29.94-61.86 | 35.43-63.43 |
| Plateletcrit (%) | 0.47±0.10 | 0.49±0.12 | 0.47±0.09 | 0.25-0.73 | 0.29-0.65 |

^*^To exclude outliers, the range limits have been defined as 2×SD above and below the mean. Where the lower limit falls below zero, the lowest observed value was used.
