# Supplementary material for: Age- and Sex-Based Hematological and Biochemical Parameters for Macaca fascicularis
Source: PLoS One. 2013 Jun 10;8(6):e64892. doi: 10.1371/journal.pone.0064892 (PMC3677909; doi:10.1371/journal.pone.0064892)
Supplement: Table S3 — Hematological values and ranges of cynomolgus monkeys aged 37–48 months. (DOC) [file pone.0064892.s003.doc]

**Table S3. Hematological values and ranges of cynomolgus monkeys aged 37-48 months.***

| **Parameter (unit)** | **Males and females (n=88)** | **Males**  **(n=16)** | **Females (n=72)** | **Male range (n=16)** | **Female range (n=72)** |
| --- | --- | --- | --- | --- | --- |
| Red blood cell (10^12^/l) | 5.78±0.43 | 5.87±0.43 | 5.76±0.44 | 5.01-6.73 | 4.88-6.64 |
| Hemoglobulin (g/l) | 130.14±8.80 | 130.31±8.00 | 130.10±9.02 | 114.31-146.31 | 112.06-148.14 |
| Hematocrit (%) | 45.78±2.98 | 45.46±2.54 | 45.85±3.09 | 40.38-50.54 | 39.67-52.03 |
| Mean corpuscular volume (fl) | 79.38±3.52 | 77.62±2.78 | 79.78±3.56 | 72.06-83.18 | 72.66-86.90 |
| Mean corpuscular hemoglobulin (pg) | 22.57±1.02 | 22.26±1.05 | 22.63±1.01 | 20.16-24.36 | 20.61-24.65 |
| Mean corpuscular hemoglobulin concentration (g/l) | 284.50±7.40 | 286.81±7.63 | 283.99±7.30 | 271.55-302.07 | 269.39-298.59 |
| Red blood cell volume distribution width-SD | 37.23±2.02 | 37.63±1.55 | 37.14±2.10 | 34.53-40.73 | 32.94-41.34 |
| Red blood cell volume distribution width-CV (%) | 13.06±0.79 | 13.49±0.62 | 12.96±0.80 | 12.25-14.73 | 11.36-14.56 |
| Reticulocyte (10^9^/l) | 56.37±22.91 | 42.73±17.09 | 59.40±23.02 | 8.55-76.91 | 13.36-105.44 |
| Reticulocyte percentage (%) | 0.97±0.38 | 0.73±0.30 | 1.03±0.38 | 0.13-1.33 | 0.27-1.79 |
| High fluorescence reticulocyte percentage (%) | 12.15±7.15 | 13.64±7.01 | 11.82±7.19 | 3.00-27.66 | 3.10-26.20 |
| Median fluorescence reticulocyte percentage (%) | 5.26±2.71 | 4.19±2.52 | 5.49±2.71 | 0.70-9.23 | 0.60-10.91 |
| Low fluorescence reticulocyte percentage (%) | 82.60±7.77 | 82.18±7.65 | 82.69±7.85 | 66.88-97.48 | 66.99-98.39 |
| Immature reticulocyte fraction (%) | 17.40±7.77 | 17.83±7.65 | 17.31±7.85 | 2.53-33.13 | 1.61-33.01 |
| White blood cell (10^9^/l) | 13.99±3.83 | 12.35±1.47 | 14.35±4.09 | 9.41-15.29 | 6.17-22.53 |
| Neutrophil (10^9^/l) | 6.55±3.69 | 4.51±2.06 | 7.00±3.83 | 0.39-8.63 | 0.66-14.66 |
| Neutrophil percentage (%) | 45.20±16.15 | 35.97±14.61 | 47.25±15.85 | 6.75-65.19 | 15.55-78.95 |
| Basophil (10^9^/l) | 0.02±0.02 | 0.02±0.01 | 0.03±0.02 | 0.01-0.03 | 0-0.07 |
| Basophil percentage (%) | 0.12±0.07 | 0.12±0.05 | 0.12±0.07 | 0.02-0.22 | 0-0.26 |
| Eosinophil (10^9^/l) | 0.38±0.35 | 0.29±0.29 | 0.40±0.36 | 0.03-0.87 | 0.03-1.12 |
| Eosinophil percentage (%) | 1.99±1.90 | 1.96±2.24 | 2.00±1.83 | 0.20-6.44 | 0.20-5.66 |
| Lymphocyte (10^9^/l) | 6.22±2.21 | 6.70±1.63 | 6.12±2.32 | 3.44-9.96 | 1.48-10.76 |
| Lymphocyte percentage (%) | 46.05±14.92 | 54.69±13.38 | 44.13±14.64 | 27.93-81.45 | 14.85-73.41 |
| Monocyte (10^9^/l) | 0.91±0.33 | 0.89±0.28 | 0.92±0.34 | 0.33-1.45 | 0.24-1.60 |
| Monocyte percentage (%) | 6.64±1.85 | 7.27±2.24 | 6.51±1.74 | 2.79-11.75 | 3.03-9.99 |
| Platelet (10^9^/l) | 373.20±102.73 | 355.25±127.38 | 377.19±97.04 | 100.49-610.01 | 183.11-571.27 |
| Mean platelet volume (fl) | 12.88±1.09 | 12.48±0.79 | 12.97±1.13 | 10.90-14.06 | 10.97-15.23 |
| Plate volume distribution width (%) | 15.93±2.31 | 15.79±2.04 | 15.96±2.38 | 11.71-19.87 | 11.20-20.72 |
| Platelet large cell ratio (%) | 47.60±7.82 | 44.20±6.02 | 48.36±8.01 | 32.16-56.24 | 32.34-64.38 |
| Plateletcrit (%) | 0.48±0.11 | 0.44±0.13 | 0.49±0.10 | 0.18-0.70 | 0.29-0.69 |

^*^To exclude outliers, the range limits have been defined as 2×SD above and below the mean. Where the lower limit falls below zero, the lowest observed value was used.
