# Supplementary material for: Age- and Sex-Based Hematological and Biochemical Parameters for Macaca fascicularis
Source: PLoS One. 2013 Jun 10;8(6):e64892. doi: 10.1371/journal.pone.0064892 (PMC3677909; doi:10.1371/journal.pone.0064892)
Supplement: Table S4 — Hematological values and ranges of cynomolgus monkeys aged 49–60 months. (DOC) [file pone.0064892.s004.doc]

**Table S4. Hematological values and ranges of cynomolgus monkeys aged 49-60 months.***

| **Parameter (unit)** | **Males and females (n=115)** | **Males**  **(n=31)** | **Females**  **(n=84)** | **Male range (n=31)** | **Female range (n=84)** |
| --- | --- | --- | --- | --- | --- |
| Red blood cell (10^12^/l) | 5.65±0.51 | 5.89±0.49 | 5.55±0.48 | 4.91-6.87 | 4.59-6.51 |
| Hemoglobulin (g/l) | 126.84±10.29 | 131.87±12.06 | 124.98±8.95 | 107.75-155.99 | 107.08-142.88 |
| Hematocrit (%) | 44.94±3.45 | 46.47±4.21 | 44.37±2.95 | 38.05-54.89 | 38.47-50.27 |
| Mean corpuscular volume (fl) | 79.80±4.02 | 78.87±3.43 | 80.14±4.18 | 72.01-85.73 | 71.78-88.50 |
| Mean corpuscular hemoglobulin (pg) | 22.52±1.24 | 22.38±1.12 | 22.57±1.28 | 20.14-24.62 | 20.01-25.13 |
| Mean corpuscular hemoglobulin concentration (g/l) | 282.41±8.05 | 284.04±7.45 | 281.81±8.22 | 269.14-298.94 | 265.37-298.25 |
| Red blood cell volume distribution width-SD | 37.74±2.58 | 37.47±2.22 | 37.84±2.71 | 33.03-41.91 | 32.42-43.26 |
| Red blood cell volume distribution width-CV (%) | 13.18±1.04 | 13.18±0.91 | 13.17±1.09 | 11.36-15.00 | 10.99-15.35 |
| Reticulocyte (10^9^/l) | 58.13±29.15 | 46.92±14.90 | 62.26±31.97 | 17.12-76.72 | 16.00-126.20 |
| Reticulocyte percentage (%) | 1.06±0.64 | 0.81±0.29 | 1.15±0.71 | 0.23-1.39 | 0.27-2.57 |
| High fluorescence reticulocyte percentage (%) | 10.73±6.31 | 11.97±5.59 | 10.27±6.53 | 3.60-23.15 | 2.00-23.33 |
| Median fluorescence reticulocyte percentage (%) | 5.07±3.37 | 3.82±2.26 | 5.54±3.60 | 0-8.34 | 0-12.74 |
| Low fluorescence reticulocyte percentage (%) | 84.20±6.99 | 84.22±5.69 | 84.20±7.45 | 72.84-95.60 | 69.30-99.10 |
| Immature reticulocyte fraction (%) | 15.80±6.99 | 15.78±5.69 | 15.81±7.45 | 4.40-27.16 | 5.00-30.71 |
| White blood cell (10^9^/l) | 13.65±2.91 | 13.52±2.16 | 13.69±3.15 | 9.20-17.84 | 7.39-22.11 |
| Neutrophil (10^9^/l) | 6.53±3.20 | 5.61±2.98 | 6.87±3.22 | 1.77-11.57 | 0.43-13.31 |
| Neutrophil percentage (%) | 46.56±15.84 | 40.10±16.48 | 48.94±15.00 | 7.14-73.06 | 18.94-78.94 |
| Basophil (10^9^/l) | 0.02±0.01 | 0.02±0.01 | 0.02±0.01 | 0.01-0.04 | 0-0.04 |
| Basophil percentage (%) | 0.11±0.05 | 0.10±0.04 | 0.12±0.06 | 0.02-0.18 | 0-0.24 |
| Eosinophil (10^9^/l) | 0.37±0.32 | 0.37±0.30 | 0.37±0.33 | 0.01-0.97 | 0.01-1.03 |
| Eosinophil percentage (%) | 2.03±1.66 | 2.05±1.54 | 2.02±1.72 | 0.10-5.13 | 0.10-5.46 |
| Lymphocyte (10^9^/l) | 5.95±1.81 | 6.69±1.87 | 5.68±1.72 | 2.95-10.43 | 2.24-9.12 |
| Lymphocyte percentage (%) | 44.91±14.67 | 50.81±15.51 | 42.73±13.82 | 19.79-81.83 | 15.09-70.37 |
| Monocyte (10^9^/l) | 0.86±0.30 | 0.92±0.33 | 0.84±0.28 | 0.26-1.58 | 0.28-1.40 |
| Monocyte percentage (%) | 6.39±2.27 | 6.94±2.76 | 6.19±2.04 | 1.42-12.46 | 2.11-10.27 |
| Platelet (10^9^/l) | 367.70±95.29 | 372.35±129.96 | 365.99±79.72 | 112.43-632.27 | 206.55-525.43 |
| Mean platelet volume (fl) | 13.00±1.08 | 12.79±1.13 | 13.07±1.05 | 10.53-15.05 | 10.97-15.17 |
| Plate volume distribution width (%) | 16.41±2.40 | 16.09±2.51 | 16.53±2.37 | 11.07-21.11 | 11.79-21.27 |
| Platelet large cell ratio (%) | 48.60±7.53 | 47.41±8.28 | 49.03±7.24 | 30.85-63.97 | 34.55-63.51 |
| Plateletcrit (%) | 0.47±0.10 | 0.47±0.13 | 0.47±0.08 | 0.21-0.73 | 0.31-0.63 |

^*^To exclude outliers, the range limits have been defined as 2×SD above and below the mean. Where the lower limit falls below zero, the lowest observed value was used.
