# Supplementary material for: Age- and Sex-Based Hematological and Biochemical Parameters for Macaca fascicularis
Source: PLoS One. 2013 Jun 10;8(6):e64892. doi: 10.1371/journal.pone.0064892 (PMC3677909; doi:10.1371/journal.pone.0064892)
Supplement: Table S5 — Hematological values and ranges of cynomolgus monkeys aged 61–72 months. (DOC) [file pone.0064892.s005.doc]

**Table S5. Hematological values and ranges of cynomolgus monkeys aged 61-72 months.***

| **Parameter (unit)** | **Males and females (n=91)** | **Males**  **(n=44)** | **Females (n=47)** | **Male range (n=44)** | **Female range (n=47)** |
| --- | --- | --- | --- | --- | --- |
| Red blood cell (10^12^/l) | 5.60±0.55 | 5.90±0.46 | 5.32±0.48 | 4.98-6.82 | 4.36-6.28 |
| Hemoglobulin (g/l) | 127.04±11.96 | 133.43±7.63 | 121.05±12.22 | 118.17-148.69 | 96.61-145.49 |
| Hematocrit (%) | 44.84±3.99 | 46.89±2.55 | 42.92±4.16 | 41.79-51.99 | 34.60-51.24 |
| Mean corpuscular volume (fl) | 80.29±4.46 | 79.76±4.10 | 80.78±4.77 | 71.56-87.96 | 71.24-90.32 |
| Mean corpuscular hemoglobulin (pg) | 22.73±1.29 | 22.69±1.11 | 22.77±1.44 | 20.47-24.91 | 19.89-25.65 |
| Mean corpuscular hemoglobulin concentration (g/l) | 283.38±7.73 | 284.74±7.50 | 282.11±7.80 | 269.74-299.74 | 266.51-297.71 |
| Red blood cell volume distribution width-SD | 37.75±3.46 | 37.55±4.05 | 37.94±2.84 | 29.45-45.65 | 32.26-43.62 |
| Red blood cell volume distribution width-CV (%) | 13.11±1.59 | 13.13±2.01 | 13.09±1.08 | 9.11-17.15 | 10.93-15.25 |
| Reticulocyte (10^9^/l) | 57.87±28.00 | 53.49±23.33 | 61.97±31.45 | 6.83-100.15 | 19.00-124.87 |
| Reticulocyte percentage (%) | 1.06±0.64 | 0.91±0.42 | 1.20±0.77 | 0.07-1.75 | 0.32-2.74 |
| High fluorescence reticulocyte percentage (%) | 10.71±5.45 | 13.04±5.74 | 8.53±4.14 | 1.56-24.52 | 0.25-16.81 |
| Median fluorescence reticulocyte percentage (%) | 5.80±3.58 | 5.33±3.06 | 6.23±3.99 | 0-11.45 | 0-16.51 |
| Low fluorescence reticulocyte percentage (%) | 83.50±5.86 | 81.63±6.28 | 85.24±4.88 | 69.07-94.19 | 75.48-95.00 |
| Immature reticulocyte fraction (%) | 16.51±5.86 | 18.37±6.28 | 14.76±4.88 | 5.81-30.93 | 5.00-24.52 |
| White blood cell (10^9^/l) | 12.75±2.19 | 13.12±2.30 | 12.40±2.04 | 8.52-17.72 | 8.32-16.48 |
| Neutrophil (10^9^/l) | 5.74±2.76 | 4.88±2.01 | 6.56±3.13 | 0.86-8.90 | 0.30-12.82 |
| Neutrophil percentage (%) | 44.84±18.39 | 37.14±14.01 | 52.04±19.19 | 9.12-51.15 | 13.66-90.42 |
| Basophil (10^9^/l) | 0.02±0.01 | 0.02±0.01 | 0.02±0.01 | 0.01-0.04 | 0-0.04 |
| Basophil percentage (%) | 0.12±0.06 | 0.12±0.06 | 0.11±0.06 | 0.10-0.24 | 0-0.23 |
| Eosinophil (10^9^/l) | 0.29±0.23 | 0.32±0.23 | 0.26±0.23 | 0.04-0.78 | 0-0.72 |
| Eosinophil percentage (%) | 1.73±1.41 | 1.84±1.47 | 1.63±1.37 | 0.30-4.78 | 0-4.37 |
| Lymphocyte (10^9^/l) | 6.00±2.24 | 7.12±2.04 | 4.95±1.90 | 3.04-11.20 | 1.15-8.75 |
| Lymphocyte percentage (%) | 47.09±17.02 | 54.38±13.03 | 40.27±17.59 | 28.32-80.44 | 5.09-75.45 |
| Monocyte (10^9^/l) | 0.78±0.30 | 0.85±0.35 | 0.71±0.22 | 0.15-1.55 | 0.27-1.15 |
| Monocyte percentage (%) | 6.22±2.13 | 6.52±2.25 | 5.94±2.00 | 2.02-11.02 | 1.94-9.94 |
| Platelet (10^9^/l) | 371.43±95.02 | 355.95±84.45 | 385.91±102.74 | 187.05-524.85 | 180.43-591.39 |
| Mean platelet volume (fl) | 12.68±1.22 | 1283±1.32 | 12.54±1.11 | 10.19-15.47 | 10.32-14.76 |
| Plate volume distribution width (%) | 15.54±2.41 | 15.74±2.67 | 15.35±2.16 | 10.40-21.08 | 11.03-19.67 |
| Platelet large cell ratio (%) | 46.45±8.70 | 47.35±9.23 | 45.60±8.19 | 28.89-65.81 | 29.22-61.98 |
| Plateletcrit (%) | 0.47±0.09 | 0.45±0.09 | 0.48±0.10 | 0.27-0.63 | 0.28-0.68 |

^*^To exclude outliers, the range limits have been defined as 2×SD above and below the mean. Where the lower limit falls below zero, the lowest observed value was used.
