# Supplementary material for: Age- and Sex-Based Hematological and Biochemical Parameters for Macaca fascicularis
Source: PLoS One. 2013 Jun 10;8(6):e64892. doi: 10.1371/journal.pone.0064892 (PMC3677909; doi:10.1371/journal.pone.0064892)
Supplement: Table S6 — Biochemical values and ranges of cynomolgus monkeys aged 13–24 months. (DOC) [file pone.0064892.s006.doc]

**Table S6. Biochemical values and ranges of cynomolgus monkeys aged 13-24 months.***

| **Parameter (unit)** | **Males and females (n=324)** | **Males**  **(n=162)** | **Females (n=162)** | **Male range (n=162)** | **Female range (n=162)** |
| --- | --- | --- | --- | --- | --- |
| Total bilirubin (μmol/l) | 1.64±0.56 | 1.64±0.56 | 1.64±0.57 | 0.52-2.76 | 0.50-2.78 |
| Total protein (g/l) | 72.37±4.93 | 72.87±4.86 | 71.87±4.95 | 63.15-82.59 | 61.97-81.77 |
| Albumin (g/l) | 41.28±4.06 | 41.45±4.42 | 41.10±3.66 | 32.61-45.87 | 33.78-48.42 |
| Globulin (g/l) | 31.10±3.60 | 31.42±3.36 | 30.77±3.80 | 24.70-38.14 | 23.17-38.37 |
| A/G | 1.34±0.20 | 1.33±0.21 | 1.36±0.20 | 0.91-1.75 | 0.96-1.76 |
| Alanine aminotransferase (IU/L) | 56.88±16.59 | 57.23±17.12 | 56.54±16.08 | 22.99-91.47 | 24.38-88.70 |
| Aspartate aminotransferase (IU/L) | 61.28±14.38 | 60.23±15.41 | 62.33±13.22 | 29.41-91.05 | 35.89-88.77 |
| Alkaline phosphatase (IU/L) | 724.76±195.58 | 697.60±197.92 | 751.91±189.97 | 301.76-1093.44 | 371.97-1131.85 |
| Gamma glutamyltransferase (IU/L) | 48.73±14.37 | 48.13±13.55 | 49.33±15.17 | 21.03-75.23 | 18.99-79.67 |
| Lactate dehydrogenase (IU/L) | 672.73±130.69 | 690.72±133.91 | 654.74±125.24 | 422.90-958.54 | 404.26-905.22 |
| Creatine kinase (IU/L) | 273.21±104.71 | 268.16±106.85 | 278.27±102.60 | 54.46-481.86 | 73.07-483.47 |
| Blood urea nitrogen (mmol/l) | 7.70±1.09 | 7.66±1.09 | 7.73±1.09 | 5.48-9.84 | 5.55-9.91 |
| Creatinine (μmol/l) | 36.19±7.10 | 37.43±7.19 | 34.94±6.80 | 23.05-51.81 | 21.34-48.54 |
| Glucose (mmol/l) | 4.19±1.29 | 4.27±1.37 | 4.11±1.22 | 1.53-7.01 | 1.67-6.55 |
| Triglyceride (mmol/l) | 0.54±0.31 | 0.57±0.38 | 0.50±0.20 | 0.14-1.33 | 0.10-0.90 |
| Total cholesterol (mmol/l) | 3.26±0.60 | 3.25±0.61 | 3.28±0.59 | 2.03-4.47 | 2.10-4.46 |
| Potassium (mmol/l) | 5.53±0.70 | 5.49±0.68 | 5.57±0.73 | 4.13-6.85 | 4.11-7.03 |
| Sodium (mmol/l) | 150.67±2.97 | 150.04±3.14 | 151.30±2.66 | 143.76-156.32 | 145.98-156.62 |
| Chloride (mmol/l) | 107.31±2.70 | 106.66±2.46 | 107.96±2.78 | 101.74-111.58 | 102.40-113.52 |
| Calcium (mmol/l) | 2.60±0.12 | 2.56±0.12 | 2.63±0.11 | 2.32-2.80 | 2.41-2.85 |
| Phosphorus (mmol/l) | 2.28±0.38 | 2.30±0.37 | 2.25±0.39 | 1.56-3.04 | 1.47-3.03 |
| Magnesium (mmol/l) | 0.92±0.09 | 0.92±0.09 | 0.93±0.09 | 0.74-1.10 | 0.75-1.11 |

^*^To exclude outliers, the range limits have been defined as 2×SD above and below the mean. Where the lower limit falls below zero, the lowest observed value was used.
