# Supplementary material for: Age- and Sex-Based Hematological and Biochemical Parameters for Macaca fascicularis
Source: PLoS One. 2013 Jun 10;8(6):e64892. doi: 10.1371/journal.pone.0064892 (PMC3677909; doi:10.1371/journal.pone.0064892)
Supplement: Table S7 — Biochemical values and ranges of cynomolgus monkeys aged 25–36 months. (DOC) [file pone.0064892.s007.doc]

**Table S7. Biochemical values and ranges of cynomolgus monkeys aged 25-36 months.***

| **Parameter (unit)** | **Males and females (n=299)** | **Males**  **(n=121)** | **Females**  **(n=178)** | **Male range (n=121)** | **Female range (n=178)** |
| --- | --- | --- | --- | --- | --- |
| Total bilirubin (μmol/l) | 1.72±0.63 | 1.68±0.55 | 1.75±0.68 | 0.58-2.78 | 0.39-3.11 |
| Total protein (g/l) | 74.47±5.43 | 73.81±6.00 | 74.91±4.97 | 61.81-85.81 | 64.97-84.85 |
| Albumin (g/l) | 41.46±4.40 | 41.01±5.30 | 41.77±3.65 | 30.41-51.61 | 34.47-49.07 |
| Globulin (g/l) | 33.00±3.78 | 32.80±3.49 | 33.14±3.96 | 25.82-39.78 | 25.22-41.06 |
| A/G | 1.28±0.20 | 1.27±0.22 | 1.28±0.20 | 0.83-1.71 | 0.88-1.68 |
| Alanine aminotransferase (IU/L) | 49.17±15.35 | 53.80±17.98 | 46.02±12.36 | 17.84-89.76 | 21.30-70.74 |
| Aspartate aminotransferase (IU/L) | 52.09±13.15 | 54.60±15.53 | 50.39±10.98 | 23.54-85.66 | 28.43-72.35 |
| Alkaline phosphatase (IU/L) | 628.57±186.65 | 597.60±181.95 | 649.62±187.36 | 233.70-961.5 | 274.90-1024.34 |
| Gamma glutamyltransferase (IU/L) | 44.63±14.94 | 46.74±13.48 | 43.20±15.73 | 19.78-73.70 | 11.74-74.66 |
| Lactate dehydrogenase (IU/L) | 571.32±130.84 | 621.89±141.88 | 536.94±110.62 | 338.13-905.65 | 315.70-758.18 |
| Creatine kinase (IU/L) | 236.33±109.50 | 228.19±86.48 | 241.87±122.64 | 55.23-401.15 | 96.00-487.15 |
| Blood urea nitrogen (mmol/l) | 7.19±1.12 | 7.36±1.14 | 7.08±1.09 | 5.08-9.64 | 4.90-9.26 |
| Creatinine (μmol/l) | 45.59±9.27 | 43.91±8.43 | 46.73±9.66 | 27.05-60.77 | 27.41-66.05 |
| Glucose (mmol/l) | 4.81±1.41 | 4.84±1.46 | 4.79±1.38 | 1.92-7.76 | 2.03-7.55 |
| Triglyceride (mmol/l) | 0.52±0.24 | 0.49±0.28 | 0.54±0.21 | 0.15-1.05 | 0.12-0.96 |
| Total cholesterol (mmol/l) | 3.33±0.74 | 3.42±0.76 | 3.27±0.73 | 1.90-4.94 | 1.81-4.73 |
| Potassium (mmol/l) | 5.65±0.68 | 5.51±0.64 | 5.74±0.69 | 4.23-6.79 | 4.36-7.12 |
| Sodium (mmol/l) | 151.67±3.74 | 150.79±4.18 | 152.28±3.29 | 142.43-159.15 | 145.70-158.86 |
| Chloride (mmol/l) | 107.5±72.76 | 106.88±2.80 | 108.03±2.64 | 101.28-112.48 | 102.75-113.31 |
| Calcium (mmol/l) | 2.63±0.16 | 2.57±0.17 | 2.67±0.14 | 2.23-2.91 | 2.39-2.95 |
| Phosphorus (mmol/l) | 2.17±0.42 | 2.26±0.43 | 2.11±0.41 | 1.40-3.12 | 1.29-2.93 |
| Magnesium (mmol/l) | 0.89±0.08 | 0.90±0.09 | 0.89±0.08 | 0.72-1.08 | 0.73-1.05 |

^*^To exclude outliers, the range limits have been defined as 2×SD above and below the mean. Where the lower limit falls below zero, the lowest observed value was used.
