# Supplementary material for: Age- and Sex-Based Hematological and Biochemical Parameters for Macaca fascicularis
Source: PLoS One. 2013 Jun 10;8(6):e64892. doi: 10.1371/journal.pone.0064892 (PMC3677909; doi:10.1371/journal.pone.0064892)
Supplement: Table S8 — Biochemical values and ranges of cynomolgus monkeys aged 37–48 months. (DOC) [file pone.0064892.s008.doc]

**Table S8. Biochemical values and ranges of cynomolgus monkeys aged 37-48 months.***

| **Parameter (unit)** | **Males and females (n=88)** | **Males**  **(n=16)** | **Females**  **(n=72)** | **Male range (n=16)** | **Female range (n=72)** |
| --- | --- | --- | --- | --- | --- |
| Total bilirubin (μmol/l) | 1.69±0.69 | 1.64±0.63 | 1.71±0.71 | 0.38-2.90 | 0.29-3.13 |
| Total protein (g/l) | 74.91±6.19 | 74.45±4.30 | 75.01±6.55 | 65.85-83.05 | 61.91-88.11 |
| Albumin (g/l) | 39.88±5.44 | 41.37±4.33 | 39.55±5.62 | 32.71-50.03 | 28.31-50.79 |
| Globulin (g/l) | 35.03±3.94 | 33.08±4.07 | 35.46±3.80 | 24.94-41.22 | 27.86-43.06 |
| A/G | 1.16±0.23 | 1.29±0.25 | 1.13±0.22 | 0.79-1.79 | 0.69-1.57 |
| Alanine aminotransferase (IU/L) | 44.65±19.45 | 43.13±10.06 | 44.99±21.02 | 23.01-63.25 | 2.95-87.03 |
| Aspartate aminotransferase (IU/L) | 47.49±10.72 | 49.25±6.80 | 47.10±11.40 | 35.65-62.85 | 24.30-69.90 |
| Alkaline phosphatase (IU/L) | 473.41±204.48 | 694.75±264.58 | 424.22±151.50 | 165.59-1223.91 | 121.22-727.22 |
| Gamma glutamyltransferase (IU/L) | 40.32±12.24 | 48.19±10.44 | 38.57±11.98 | 27.31-69.07 | 14.61-62.53 |
| Lactate dehydrogenase (IU/L) | 518.97±141.17 | 518.50±125.48 | 519.07±145.24 | 267.54-769.46 | 228.59-809.55 |
| Creatine kinase (IU/L) | 255.70±146.82 | 259.06±93.78 | 254.96±156.70 | 71.50-446.62 | 105.00-568.36 |
| Blood urea nitrogen (mmol/l) | 6.83±1.18 | 6.89±0.91 | 6.82±1.23 | 5.07-8.71 | 4.36-9.28 |
| Creatinine (μmol/l) | 52.00±9.91 | 54.39±6.73 | 51.47±10.45 | 40.93-67.85 | 30.57-72.37 |
| Glucose (mmol/l) | 4.91±1.36 | 4.34±0.68 | 5.04±1.44 | 2.98-5.70 | 2.16-7.92 |
| Triglyceride (mmol/l) | 0.73±0.72 | 0.71±0.44 | 0.74±0.76 | 0.17-1.59 | 0.13-2.26 |
| Total cholesterol (mmol/l) | 3.39±0.67 | 3.11±0.58 | 3.45±0.67 | 1.95-4.27 | 2.11-4.79 |
| Potassium (mmol/l) | 5.53±0.76 | 5.50±0.77 | 5.53±0.76 | 3.96-7.04 | 4.01-7.05 |
| Sodium (mmol/l) | 152.43±3.64 | 153.38±2.73 | 152.22±3.79 | 147.92-158.84 | 144.64-159.80 |
| Chloride (mmol/l) | 108.11±2.77 | 107.50±2.48 | 108.25±2.83 | 102.54-112.46 | 102.59-113.91 |
| Calcium (mmol/l) | 2.64±0.15 | 2.63±0.10 | 2.64±0.16 | 2.43-2.83 | 2.32-2.96 |
| Phosphorus (mmol/l) | 2.05±0.45 | 2.17±0.39 | 2.02±0.46 | 1.39-2.95 | 1.10-2.94 |
| Magnesium (mmol/l) | 0.86±0.09 | 0.84±0.06 | 0.87±0.10 | 0.72-0.96 | 0.67-1.07 |

^*^To exclude outliers, the range limits have been defined as 2×SD above and below the mean. Where the lower limit falls below zero, the lowest observed value was used.
