# Supplementary material for: Age- and Sex-Based Hematological and Biochemical Parameters for Macaca fascicularis
Source: PLoS One. 2013 Jun 10;8(6):e64892. doi: 10.1371/journal.pone.0064892 (PMC3677909; doi:10.1371/journal.pone.0064892)
Supplement: Table S9 — Biochemical values and ranges of cynomolgus monkeys aged 49–60 months. (DOC) [file pone.0064892.s009.doc]

**Table S9. Biochemical values and ranges of cynomolgus monkeys aged 49-60 months.***

| **Parameter (unit)** | **Males and females (n=115)** | **Males**  **(n=31)** | **Females**  **(n=84)** | **Male range (n=31)** | **Female range (n=84)** |
| --- | --- | --- | --- | --- | --- |
| Total bilirubin (μmol/l) | 1.86±0.65 | 1.78±0.58 | 1.90±0.67 | 0.62-2.94 | 0.56-3.24 |
| Total protein (g/l) | 76.47±6.57 | 75.70±8.59 | 76.75±5.68 | 58.52-92.88 | 65.39-88.11 |
| Albumin (g/l) | 39.87±5.15 | 39.85±7.27 | 39.87±4.16 | 25.31-54.39 | 31.55-48.19 |
| Globulin (g/l) | 36.60±4.58 | 35.86±4.32 | 36.88±4.66 | 21.32-44.50 | 27.56-46.20 |
| A/G | 1.10±0.19 | 1.12±0.22 | 1.09±0.18 | 0.68-1.56 | 0.73-1.45 |
| Alanine aminotransferase (IU/L) | 44.70±18.57 | 44.52±16.68 | 44.77±19.31 | 11.16-77.88 | 6.15-83.39 |
| Aspartate aminotransferase (IU/L) | 45.56±13.00 | 47.06±11.95 | 45.00±13.40 | 23.16-70.96 | 18.20-71.80 |
| Alkaline phosphatase (IU/L) | 361.69±175.30 | 547.19±121.32 | 293.23±138.89 | 304.55-789.83 | 100.00-571.01 |
| Gamma glutamyltransferase (IU/L) | 37.04±10.73 | 41.23±11.80 | 35.50±9.95 | 17.63-64.83 | 15.60-55.40 |
| Lactate dehydrogenase (IU/L) | 497.57±133.14 | 533.03±131.99 | 484.48±131.94 | 269.05-797.01 | 220.60-748.36 |
| Creatine kinase (IU/L) | 267.59±248.00 | 265.97±145.60 | 268.19±277.15 | 101.00-557.17 | 92.00-822.49 |
| Blood urea nitrogen (mmol/l) | 6.29±1.01 | 6.38±1.02 | 6.26±1.01 | 4.34-8.42 | 4.24-8.28 |
| Creatinine (μmol/l) | 59.57±11.04 | 64.76±11.20 | 57.66±10.41 | 42.36-87.16 | 36.84-78.48 |
| Glucose (mmol/l) | 5.00±1.69 | 4.68±1.27 | 5.12±1.82 | 2.14-7.22 | 1.48-8.76 |
| Triglyceride (mmol/l) | 0.58±0.35 | 0.61±0.49 | 0.57±0.29 | 0.09-1.59 | 0.23-1.15 |
| Total cholesterol (mmol/l) | 3.24±0.69 | 3.19±0.61 | 3.27±0.71 | 1.97-4.41 | 1.85-4.69 |
| Potassium (mmol/l) | 5.76±0.69 | 5.68±0.66 | 5.79±0.70 | 4.36-7.00 | 4.39-7.19 |
| Sodium (mmol/l) | 153.25±3.60 | 152.94±3.59 | 153.37±3.61 | 145.76-160.12 | 146.15-160.59 |
| Chloride (mmol/l) | 107.65±2.97 | 105.87±2.36 | 108.31±2.91 | 101.15-110.59 | 102.49-114.13 |
| Calcium (mmol/l) | 2.67±0.17 | 2.63±0.17 | 2.69±0.16 | 2.29-2.97 | 2.37-3.01 |
| Phosphorus (mmol/l) | 2.04±0.45 | 2.17±0.37 | 2.00±0.46 | 1.43-2.91 | 1.08-2.92 |
| Magnesium (mmol/l) | 0.87±0.08 | 0.84±0.07 | 0.89±0.08 | 0.70-0.98 | 0.73-1.05 |

^*^To exclude outliers, the range limits have been defined as 2×SD above and below the mean. Where the lower limit falls below zero, the lowest observed value was used.
