# Supplementary material for: Age- and Sex-Based Hematological and Biochemical Parameters for Macaca fascicularis
Source: PLoS One. 2013 Jun 10;8(6):e64892. doi: 10.1371/journal.pone.0064892 (PMC3677909; doi:10.1371/journal.pone.0064892)
Supplement: Table S10 — Biochemical values and ranges of cynomolgus monkeys aged 61–72 months. (DOC) [file pone.0064892.s010.doc]

**Table S10.** **Biochemical values and ranges of cynomolgus monkeys aged 61-72 months.***

| **Parameter (unit)** | **Males and females (n=91)** | **Males**  **(n=44)** | **Females**  **(n=47)** | **Male range (n=44)** | **Female range (n=47)** |
| --- | --- | --- | --- | --- | --- |
| Total bilirubin (μmol/l) | 1.65±0.48 | 1.59±0.44 | 1.70±0.51 | 0.71-2.47 | 0.68-2.72 |
| Total protein (g/l) | 78.24±6.44 | 79.28±5.57 | 77.26±7.09 | 68.14-90.42 | 63.08-91.44 |
| Albumin (g/l) | 40.18±5.63 | 42.15±3.62 | 38.34±6.53 | 34.91-49.39 | 25.28-51.40 |
| Globulin (g/l) | 38.06±5.12 | 37.13±4.49 | 38.92±5.55 | 28.15-46.11 | 27.82-50.02 |
| A/G | 1.09±0.21 | 1.16±0.17 | 1.01±0.23 | 0.82-1.50 | 0.55-1.47 |
| Alanine aminotransferase (IU/L) | 45.65±24.11 | 42.59±16.93 | 48.51±29.19 | 8.73-76.45 | 12.00-106.89 |
| Aspartate aminotransferase (IU/L) | 44.20±15.84 | 46.32±12.63 | 42.21±18.27 | 21.06-71.58 | 5.67-78.75 |
| Alkaline phosphatase (IU/L) | 332.69±179.68 | 422.57±192.47 | 248.55±116.51 | 37.63-807.51 | 15.53-481.57 |
| Gamma glutamyltransferase (IU/L) | 37.76±11.95 | 38.18±8.88 | 37.36±14.33 | 20.42-55.94 | 8.70-66.02 |
| Lactate dehydrogenase (IU/L) | 449.01±107.57 | 454.00±114.69 | 444.34±101.49 | 224.62-683.38 | 241.36-647.32 |
| Creatine kinase (IU/L) | 240.13±125.58 | 232.84±113.95 | 246.96±136.46 | 94.00-460.74 | 60.00-519.88 |
| Blood urea nitrogen (mmol/l) | 6.24±1.11 | 6.12±0.94 | 6.34±1.25 | 4.24-8.00 | 3.84-8.84 |
| Creatinine (μmol/l) | 67.99±15.88 | 76.82±17.11 | 59.73±8.68 | 42.60-111.04 | 42.37-77.09 |
| Glucose (mmol/l) | 5.29±1.77 | 5.56±1.68 | 5.03±1.83 | 2.20-8.92 | 1.37-8.69 |
| Triglyceride (mmol/l) | 0.56±0.28 | 0.53±0.27 | 0.58±0.30 | 0.16-1.07 | 0.13-1.18 |
| Total cholesterol (mmol/l) | 3.08±0.80 | 2.94±0.58 | 3.20±0.95 | 1.78-4.10 | 1.30-5.10 |
| Potassium (mmol/l) | 6.08±0.71 | 6.37±0.81 | 5.82±0.47 | 4.75-7.99 | 4.88-6.76 |
| Sodium (mmol/l) | 154.57±3.98 | 155.77±4.32 | 153.45±3.30 | 147.13-164.41 | 146.85-160.05 |
| Chloride (mmol/l) | 107.52±2.80 | 107.45±2.81 | 107.57±2.82 | 101.83-113.07 | 101.93-113.21 |
| Calcium (mmol/l) | 2.68±0.17 | 2.73±0.16 | 2.64±0.16 | 2.41-3.05 | 2.32-2.96 |
| Phosphorus (mmol/l) | 2.18±0.49 | 2.36±0.52 | 2.01±0.39 | 1.32-3.40 | 1.23-2.79 |
| Magnesium (mmol/l) | 0.89±0.08 | 0.90±0.08 | 0.87±0.08 | 0.74-1.06 | 0.71-1.03 |

^*^To exclude outliers, the range limits have been defined as 2×SD above and below the mean. Where the lower limit falls below zero, the lowest observed value was used.
